# “Crowdsourcing” ten years in: A review

**DOI:** 10.7189/jogh.07.020601

**Published:** 2017-12

**Authors:** Kerri Wazny

**Affiliations:** Centre for Global Health, Usher Institute for Informatics and Population Sciences, University of Edinburgh, Edinburgh, Scotland, UK

## Abstract

**Background:**

First coined by Howe in 2006, the field of crowdsourcing has grown exponentially. Despite its growth and its transcendence across many fields, the definition of crowdsourcing has still not been agreed upon, and examples are poorly indexed in peer–reviewed literature. Many examples of crowdsourcing have not been scaled–up past the pilot phase. In spite of this, crowdsourcing has great potential, especially in global health where resources are lacking. This narrative review seeks to review both indexed and grey crowdsourcing literature broadly in order to explore the current state of the field.

**Methods:**

This is a review of reviews of crowdsourcing. Semantic searches were conducted using Google Scholar rather than indexed databases due to poor indexing of the topic. 996 articles were retrieved, of which 69 were initially identified as being reviews or theoretically–based. 21 of these were found to be irrelevant and 48 articles were reviewed.

**Results:**

This narrative review focuses on defining crowdsourcing, taxonomies of crowdsourcing, who constitutes the crowd, research that is amenable to crowdsourcing, regulatory and ethical aspects of crowdsourcing and some notable examples of crowdsourcing.

**Conclusions:**

Crowdsourcing has the potential to be hugely promising, especially in global health, due to its ability to collect information rapidly, inexpensively and accurately. Rigorous ethical and regulatory controls are needed to ensure data are collected and analysed appropriately and crowdsourcing should be considered complementary to traditional research methods.

“No one knows everything, everyone knows something [and] all knowledge resides in humanity; digitalisation and communication technologies must become central in this coordination of far flung genius” [1]. Although examples of crowdsourcing and “wisdom of the crowds” have been reported hundreds of years ago [2,3], the term “crowdsourcing” was coined in 2006 by Howe in his *Wired* magazine article [4]. In the article, Howe defines crowdsourcing as “the act of a company or institution taking a function once performed by employees and outsourcing it to an undefined (and generally large) network of people in the form of an open call” [4] and he adds that “crowdsourcing is the mechanism by which talent and knowledge is matched to those in need of it” to the definition in a later article [5]. Since Howe’s article and partially due to the availability of modern technology [6,7], use of crowdsourcing has skyrocketed [8]. Although research in this area has grown exponentially in the last decade, many authors feel that the potential of crowdsourcing is still underutilised and underexploited [5,9–11].

As crowdsourcing requires, depending on the definition, ‘outsourcing’ a task or tasks to a large crowd [12], advances in technology have facilitated the efficiency of this method [2,6,13–15]. Indeed, research that was previously inconceivable due to the scale is now achievable through crowdsourcing [6]. Kamajian states that 35% of smart phone users check their phones prior to getting out of bed and, as of 2013, over 5 billion people worldwide had access to mobile phones [14,16]. Prior to Howe’s *Wired* article, Louis van Ahn introduced the idea of human computing, where humans are used to solve complex problems that computers are not capable of [17]. While machine learning has made great strides, computers are poor at perception; humans can conceptualise, discriminate and filter, learn, adapt using their background knowledge and apply common sense and experience that machine are unable to do [18]. In addition to humans actively crowdsourcing data, ubiquitous computing, where computers exist through the physical environment, are virtually invisible to the user, and act as passive sensors has great potential for generating large amounts of data [16,18]. Cell phones, for example, can collect photo, video, acoustic, gyroscopic, acccelerometric and proximal information and can also be used to add pairing devices to collect additional information, such as pollution sensors [16]. Crowdsourced spatial analysis from GIS data can be very useful, especially for providing resources in emergency situations, for delivering logistics and for efficient targeting of interventions [19].

As individuals are biased towards the correct answer, Buecheler et al. estimate that if a million individuals were to contribute towards answering a problem via crowdsourcing, there would be a 97.7% likelihood that the crowd would arrive at the correct answer [20]. While pilot studies have not reached sample sizes close to that scale, many have had great success in achieving extremely promising results. For example, crowdsourcing has been demonstrated to produce accurate results across a range of medical diagnostic studies, including malaria, grading images for glaucoma and diabetic retinopathy, skin self–examination for skin cancers, and images for cancer polyps [21–26].

Despite the interest in the area of crowdsourcing exploding in the past decade, many authors do not agree on its definition or on what counts as crowdsourcing, with some academics considering Wikipedia a “classic” example of crowdsourcing, for example, while others insist it is not crowdsourcing [12]. Text and data mining is another example that is on the fringe of crowdsourcing’s definition.

In addition to there being many definitions of crowdsourcing, many authors have offered different taxonomies of crowdsourcing, some focusing on types of crowdsourcing while others focus on its production model. Furthermore, there are debates on who participates in crowdsourcing – whether it is laypersons, amateurs, professionals, experts, or a combination.

Although crowdsourcing has existed for decades, it is agreed upon that technology has facilitated its growth. Platforms such as Amazon Mechanical Turk and Crowdflower enable companies to hire workers to perform crowdsources exercises for extremely low prices. Other crowdsourcing platforms, such as Innocentive or Crowdmed, offer a competitive winner–takes–all model. Sensors in wearable technology have also facilitated the ability to collect mass amounts of information.

Crowdsourcing can increase the accuracy of computer automated tasks, lower costs, increase the scale of research, transcend boundaries and borders, produce novel discoveries and increase the speed of research progression, among other benefits. However, there are concerns with the generalisability of the samples, as the crowd is self–selected, security and data protection issues of sensitive data, and the possibility of malicious workers. Some studies have added quality protection measures to weed out malicious workers, such as adding cut–offs for scores on previous tasks and screening questions. Additional regulation is needed for ethical issues, such as obtaining informed consent and data use policies.

Crowdsourcing has considerable benefits in research, as it has the potential to substantially lower costs while massively increasing the sample size and researchers can receive the data in real–time [7,16,19,27–29]. Because of these qualities, crowdsourcing has potential to improve global health research. Indeed, crowdsourcing is used frequently to set research priorities in global health, most often in maternal, newborn and child health, due to the popularity of the Child Health and Nutrition Research Initiative’s (CHNRI) method of research priority setting which uses collective opinion to identify and score research priorities against a set list of criteria [30]. The CHNRI method is becoming the most frequently used research priority setting method due to its transparent, systematic nature; it was designed to capitalise on the principles of Surowiecki’s “Wisdom of the Crowd,” which will be described in the further in the paper [31]. Furthermore, research in global health faces an even larger burden than research in high–income countries with regards to funding, logistics, poor existing health care systems, health care workers to collect data, equipment, and patient access to health care, especially in rural or conflict areas [21,32–37]. As access to mobile phones in low– and middle–income countries is still increasing, crowdsourcing may provide a complementary route of data collection to traditional sources, capitalising on structures and knowledge already in place in the countries [38].

## METHODS

As previous authors had reported few search results in indexed journals [10,27,39] and crowdsourcing is a new method, semantic searches in Google Scholar were used to retrieve both peer–reviewed and grey literature published on crowdsourcing. “Crowdsourcing” as well as ‘crowdsourcing’ joint with health terms, such as genetics, diagnosis, epidemiology, surveillance, public health and disease were searched in August, 2015. Crowdsourcing and global health was searched initially, as well, but the results overlapped entirely with crowdsourcing and health and crowdsourcing and public health. The titles of results were scanned until it was clear that results appearing were no longer relevant. Full details of the searches, as well as the number of pages of Google Scholar results scanned, can be found in **Box 1**. In total, 995 results were identified through the Google Scholar search, which is substantially more than any other reviews have identified. 375 results were discarded as duplicates or irrelevant once abstracts were read.

Box 1 **Crowdsourcing Semantic Searches Conducted in Google Scholar**

1. “Crowdsourcing”
  - Up to 25 pages
2. “Crowdsourcing” and “Health”
  - Up to 15 pages
3. “Crowdsourcing” and “Immunology”
  - Up to 5 pages
4. “Crowdsourcing” and “Genetics”
  - Up to 9 pages
5. “Crowdsourcing” and “Public Health”
  - Up to 20 pages
6. “Crowdsourcing” and “Disease”
  - Up to 25 pages
7. “Crowdsourcing” and “Surveillance”
  - To 20 pages
8. “Crowdsourcing” and “Diagnosis”
  - Up to page 14

Results were organised within Endnote into categories, including reviews, theory of crowdsourcing, health, public planning, GPS–related, translation, robotics, visual perception, logistics of crowdsourcing, which was broken down into motivations, quality, reliability, stability, and others. This review reports on the papers reporting on reviews and theory as well as a portion of the health–related papers, as there were 285 health papers and many of their interventions overlapped. Further reviews can be conducted with the results of the search and organised Endnote library, but are outside the scope of the current review.

The reviews and theoretical papers generally covered the varying definitions of crowdsourcing, taxonomies of crowdsourcing, participants, modes of participation, when research is suitable for crowdsourcing, benefits and concerns with crowdsourcing, recommendations for regulation and quality control, including ethical regulations and examples of crowdsourcing.

## DISCUSSION

### Defining crowdsourcing

The definition of crowdsourcing as well as some ‘traditional’ examples of crowdsourcing, such as Wikipedia, are highly debated; this is likely due to both the relative newness of the term and the flexibility and adaptability of the method [1,5,7,8,10–12,20,40–43]. To further complicate authors’ attempts to define crowdsourcing, there are a variety of related concepts that have been used synonymously, including: citizen science, health 2.0, wisdom of the crowds, peer production, open sourcing, expert sourcing, collective intelligence, human computation, community–based participatory research, participatory epidemiology, outsourcing and open sourcing [1,3,7,12,43]. While some, like expert–sourcing, are easy to understand as crowdsourcing with experts, the differences between crowdsourcing and others are more nuanced.

Three terms, specifically, are used abundantly in literature and often interchangeably with crowdsourcing: health 2.0, wisdom of the crowds, and citizen science. While applications of crowdsourcing are often a combination of these, especially in the field of health, there are important distinctions between them [5,8,11].

Swan defines citizen science as non–professionals conducting science–related activities [8]. Non–professionals can include scientists of professionals who are conducting activities outside their own fields (so that they are amateurs in that field). All of the examples given by Swan include citizen science at a mass–scale, and thus are all citizen–science activities that are also using crowds [8]. It may be possible to imagine an activity in which citizens are acting as scientists, collecting data or participating in an experiment that is not at a mass scale, however, such as if citizens provide feedback in the design of a study at a small–scale. Therefore, not all citizen science must be crowdsourcing, but much of it will be.

Health 2.0 is defined, also by Swan, as active participation in one’s health care using web 2.0 technologies [8]. This could include using m–Health applications to track diet and exercise, for example. Using these applications itself would not be considered crowdsourcing, as data are not necessarily collected and there is no unified output. However, if data were collected, the act of collecting data from this could be considered crowdsourcing. Thus, health 2.0 technology can contribute towards crowdsourcing but is not necessarily crowdsourcing.

“Wisdom of the crowds” is another related term. This refers to the use of *knowledge* of a large crowd of people and also requires an intelligent crowd. This also differs, slightly, from the term crowdsourcing, as not all crowdsourcing tasks require knowledge or intelligence. Unlike citizen science and health 2.0, all ‘wisdom of the crowds’ tasks are forms of crowdsourcing, but not all crowdsourcing are necessarily applications of a ‘wisdom of the crowds.’ An example of a task requiring intelligence would be using a crowd to diagnose malaria cells in blood smears. In this, each participant needs to use their knowledge or intelligence to consider which blood smears contain or do not contain malaria parasites. Some, perhaps arguable, examples of crowdsourcing that would *not* be considered requiring knowledge could be RECAPTCHA, passive surveillance such as environmental surveillance using ubiquitous computing and mobile phones, reporting systems, or text mining. In his book, Surowiecki lists four requirements for an intelligent crowd that are particularly important for crowdsourcing tasks that require knowledge (ie, are ‘wisdom of the crowds’ tasks). They are: (i) diversity, which adds perspectives that would otherwise be absent; (ii) independence, limiting the influence of one person’s opinions on other’s; (iii) decentralisation, to develop tacit, specialised knowledge; and (iv) aggregation, to combine the diverse, independent, knowledgeable opinions of the crowd [31].

In addition to these three terms, crowdsourcing is often contrasted to open sourcing or outsourcing. Although some authors believe that crowdsourcing is a special form of outsourcing [3], many authors conclude that the major difference between crowdsourcing and outsourcing is the presence of a contract [10]. In addition, in a crowdsourcing exercise, the organisation or crowdsourcing initiator has the rights to whatever is produced and the crowd is aware of this [10]. Intellectual property rights are also one of the major differences between crowdsourcing and open sourcing or peer–production, along with the hierarchical structure of crowdsourcing [1,10]. In open–sourcing or peer–production, the product that is being worked on is free, will remain free and the crowd that is working on it volunteers their labour to make the free product better. In crowdsourcing, the crowd is volunteering but, if they are contributing to a product, it is unlikely to be available for free [1]. Furthermore, with open–sourced and peer–production models, which are usually software, the software and its code are released and coders work and submit bug fixers as they come up, with no hierarchy. With crowdsourcing, there is a clear call for work.

Crowdsourcing has other key features including a clear, open call for participants and a large crowd. Since there are many different definitions, Estelles–Arolas et al. reviewed definitions of crowdsourcing and developed an integrative definition using Tatarkiewicz’s approach, which is based on developing a global definition of the concept of art. In their review, the authors found 8 key qualities of a crowdsourcing definition, namely: a) who forms the crowd; b) what the crowd has to do; c) how the crowd is reimbursed; d) who initiates the crowdsourcing process; e) what the product of crowdsourcing is; f) what type of process is used; g) what type of call is used; and h) by what medium the call is made [12].

The integrative definition that the authors devise from their review is [12]:

“Crowdsourcing is a type of participative online activity in which an individual, and institution, a non–profit organisation, or company proposes to a group of individuals of varying knowledge, heterogeneity, and number, via a flexible open call, the voluntary undertaking of a task. The undertaking of the task, of variable complexity and modularity, and in which the crowd should participate bringing their work, money, knowledge and/or experience, always entails mutual benefit. The user will receive the satisfaction of a given type of need, be it economic, social recognition, self–esteem, or the development of individual skills, while the crowdsourcer will obtain and utilize to their advantage what the user has brought to the venture, whose form will depend on the type of activity undertaken.”

Although he describes it as a taxonomy rather than a definition, the features of Geiger et al.’s description of crowdsourcing is similar to Estelles–Arolas et al.’s integrative definition. The key features Geiger describes are: (i) pre–selection of contributors (how ‘open’ the call is, but usually the authors state there are no limits); (ii) accessibility of peer contributors (whether they can access each other’s contributions); (iii) aggregation (to what extent the input is used); and (iv) remuneration (fixed, success–based or none) [12,44].

Each feature of Estelles–Arolas et al.’s integrative definition and Geiger et al.’s taxonomy is discussed below.

### Estelles–Arolas et al.’s and Geiger et al.’s

#### Who forms the crowd (corresponds to Geiger et al.’s pre–selection of contributors)

The majority of authors reviewed by Estelles–Arolas et al. did not provide a distinct definition for their crowds, instead describing a crowd as a large group of people or individuals, consumers, or volunteers [12]. The authors found that the crowds size could vary from a few thousand to several hundred thousand and their skill levels could also vary from being very unskilled, in the case of Amazon Mechanical Turk (AMT) workers to extremely skilled InnoCentive submitters, who are often hold PhDs [12]. However, in a study in business management, ideas generated by professionals and laypeople through crowdsourcing were compared and those by laypeople were more novel and offered the customer more benefits; however they were less feasible [14].

In contrast, Brabham specifically examined how authors refer to crowds and found that the majority of articles refer to crowds as being composed of amateurs [7]. However, he argues that ‘amateurism’ in crowdsourcing is a myth, and blames this partially on Howe’s original definition of crowdsourcing. In Brabham’s review, he found that most crowds were comprised of self–selected professionals, such as InnoCentive’s submitters being extremely well–educated, those who submitted advertisements for Doritos’ SuperBowl advertisement contest were mostly film school students and the majority of iStock Photos’ submitters are professional photographers [7]. Is amateurism not being paid or lacking access to tools? Brabham cites Stebbins’ definition of amateurs: “amateurs are guided by standards of excellence set by professionals and not necessarily inferior, feel an obligation to their pursuit, restrain professions from over–emphasising technique and from stressing superficialities instead of meaningful or profound work or products” [7]. He contrasts this definition with a definition of amateurs as “one lacking experience” and further argues that professionalism is a class about status and linked to capitalism. Crowdsourcing, then, represents the ‘race to the bottom’ to allow greater profit margins by falsely positioning who should be described as professionals as amateurs and underpaying them for their work [7].

With regards to the demographics of the crowd, Ranard et al.’s review found that few articles reported on demographics and for those that did, the level of demographics reported varied [27]. However, Khare et al. state that the crowd should be poorly defined and diverse [3]. Brabham believes that there are three types of diversity necessary: (i) identity; (ii) skills; and (iii) political investment. However, his vision of identity includes national, sex, gender, race, economic class, disability, religion, among other things [7]. As Surowiecki stated, diversity is important to having a wise crowd [14,31]. Kamajian found that technical and ‘social marginality’ were beneficial for success in InnoCentive submissions; social marginality was defined as being female [14].

Geiger et al. aim to classify different types of crowdsourcing processes, and in doing so describe the ‘openness’ of their calls. The authors found that most crowdsourcing processes have a completely open call but some restrict contributions from participants by using either qualification–based (ie, the contributors need to have demonstrated a certain level of qualification or skills prior to participating) or context–based (ie, the participants need to be in a certain demographic) limitations [44].

#### What the crowd has to do

Estelles–Arolas et al.’s review came across a dichotomy regarding the purpose of the crowd; one group of authors believed that the purpose of the crowd was to complete tasks and the other, to solve problems [12]. Some authors believe that tasks must be divisible into lower–level tasks in order to be suitable for crowdsourcing [5,12,28]. Estelles–Arolas et al. conclude that “any non–trivial problem can benefit from crowdsourcing” [12].

In this review, various authors attempted to make classifications of *what* crowdsourcing should aim to do. These are found in **Table 1**. As one can see, some authors disagree that open innovation and peer production fall outside the realm of crowdsourcing. The authors also differ with regards to the level of detail of their classifications, ranging from a dichotomous classification of microtasks and megatasks [3] to Geiger et al.’s and Saxton et al.’s more detailed classifications of types of crowdsourcing processes [11,44]. However, at its heart, many of the classifications can be conflated to combination of Geiger et al.’s second and Aitamurto et al.’s classifications: crowd creation; crowd voting (including prediction markets); crowd processing; crowd rating; crowd solving; and crowd funding. However, crowd funding is the mobilisation of monetary funds for a common goal and thus is not covered by this review [44,45].

**Table 1:** Classifications of what crowdsourcing should aim to do

Prpic [39]	Public health; health promotion; health maintenance; and health research
Kamajian [14]	Collection; collaboration; individual decisions; and group decisions
Brabham et al. [9]	Knowledge discovery and management (gathering, organising and reporting); distributed human intelligence tasking problems (ie, AMT); broadcast search (ie, locating a needle in a haystack, Innocentive); and peer–vetted creative problem production (ie, Threadless)
Aitamurto et al. [45] (citing Howe)	Crowd wisdom; crowd creation; crowd voting (including prediction markets); and crowd funding
Saxton et al. [11]	Intermediate (find, finish and earn through the web, ie, AMT); citizen media production; collaborative software development; digital goods sales (ie, iStock Photo); peer–to–peer social financing (ie, Kickstarter); product design (ie, Threadless); consumer reporting; knowledge base building; collaborative science projects
Khare et al. [3]	Microtasks (disseggregated then joined); and mega–tasks (“open innovation”)
Parvanta et al. [46]	Crowdfunding; crowd labour; and crowd research
Pedersen et al. [47]	Co–creation; crowd creation; crowd voting; crowd wisdom; and crowd funding
Yuen et al. [17]	Voting systems; information sharing; games; and creative
Geiger et al. (a) [44]	Integrative sourcing without remuneration (ie, Wikis); selective sourcing without crowd assessment (ie, private contributors, public design or innovation contests); selective sourcing with crowd assessment (contests where the public assesses contributions); integrative sourcing with success–based remuneration (ie, iStock Photo); and integrative sourcing with fixed remuneration
Geiger et al. (b) [44]	Crowd processing (ie, GalaxyZoo); crowd rating (ie, Trip Advisor); crowd solving (ie, FoldIt); and crowd creation (ie, Threadless)

#### How the crowd is reimbursed (corresponds to Geiger et al.’s remuneration)

Many of the authors in Estelles–Arolas et al.’s review identified reimbursement as monetary reimbursement. The range of monetary reimbursement is large, varying from US$ 0.01 for each human intelligence task (HIT) performed on the AMT platform to millions of dollars for the successful solution chosen from InnoCentive’s competitions [12]. Geiger et al. look at whether reimbursement is fixed, varied or voluntary as a means to classify crowdsourcing projects. AMT projects would have fixed reimbursements, where all members of the crowd are remunerated the same amount for their participation, whereas InnoCentive employs a success–based remuneration plan [44]. However, both Estelles–Arolas et al. and Geiger et al. acknowledge that not all crowdsourcing projects pay monetarily, and that monetary remuneration is not necessarily the primary motivation for the participants. Estelles–Arolas et al.’s review suggests that participant motivations mirror Maslow’s hierarchy of individual needs: economic reward, social recognition, self–esteem and development of individual skills. In addition to or in lieu of financial rewards, individuals participating in crowdsourcing are able to develop their skills through freelancing, contribute to their community, have fun, share knowledge and be recognised through their contributions, Parvanta et al. describe the motivations as the ‘four f’s:’ fun, fulfilment, fame, and fortune [46]. In addition to these, crowdsourcing activities such as RECAPTCHA have capitalised on task being integral to another task the user is trying to access and have been wildly successful [6]. An additional, similar, motivation that Swan identifies is biocitizenry, in which the crowd participates in order to gain access to studies [8]. Doan and colleagues suggest that, in addition to those listed above, making users pay for a service, providing ownership situations or requiring contribution to crowdsourcing through their employment, having instant gratification or providing an enjoyable experience of a necessary service will motivate a crowd [43]. In their review, Zhao and Zhu found that only 2/55 studies used motivational theories in designing their interventions [10]. Zhao and Zhu, Kostkova, and Kittur call for further research into crowd motivation, specifically the use of serious gaming, auction bidding and understanding crowd behaviour in task selection [10,38,48].

Some authors reviewed mentioned inequities regarding crowd contributions. Parvanta et al. describe a 90%/9%/1% rule for participation, in which 90% of the crowd observes, 9% participates from time–to–time and 1% participates regularly [46]. This breakdown would be more amenable to a service such as YouTube or Wikipedia, where observing or viewing a product is an option. Zhao and Zhu describe super contributors, contributors and outliers but do not give a percentage of contributions between the three categories [10]. Holley states that the majority of work is completed by 10% of the crowd and these super contributors are often retirees or young, dynamic professionals [49].

#### Who initiates the crowdsourcing process

Generally, an institution or organisation initiates the crowdsourcing process with an open call [12]. However, there have also been instances where the crowdsourcer has been a governmental department, such as in Iceland [42].

#### What the product of crowdsourcing is

Many authors reviewed by Estelles–Arolas et al. felt that the initiator receives the result sought for the task advertised, which was usually the result for a given problem. Others believed the product was either knowledge, ideas, or some type of added value [12]. The exact type product of crowdsourcing can be very diverse and has not been agreed upon, but generally is some type of result that is requested by and has value to the initiator.

#### What type of process is used

Estelles–Arolas et al.’s review found many authors who identified crowdsourcing as an outsourcing process, specifically referring to AMT while others referred to it as a problem–solving process or a production model [12]. As described previously, crowdsourcing differs from open sourcing, outsourcing and peer–production. Many articles reviewed in this review specifically mentioned the use of online, outsourcing–like mediums, such as AMT [3,6,48] and CrowdFlower [6]. In AMT and CrowdFlower, the initiator (or crowdsourcer) posts a task and the ‘crowd’ responds and are paid in small quantities for completing small HITs. Other online platforms use distributed online processes to compete for the best solution, such as InnoCentive or CrowdMed [27,32]. Advances in mHealth, such as wearable technologies and sensors, could enable real–time data collection and monitoring from mass amounts of people [38]. Kostkova estimates that 75 million wearable technological devices will have been shipped by 2018 and calls for behavioural research using these devices [38]. The data from these devices could be considered crowdsourcing if there is a specific call for data. Gamification has also been used to enhance the crowd’s experience while crowdsourcing and encourage participation [21,50,51]. Finally, another debatable form of crowdsourcing could be data mining, using Twitter posts or Google Flu Trends [32,52,53]. However, according to the definitions of crowdsourcing by both Estelles–Arolas et al. and Geiger et al., data mining would not be in the realm of crowdsourcing.

#### What type of call is used

The majority of authors reviewed by Estelles–Arolas et al. refer to an open call as the form of call that must be made in order to satisfy a crowdsourcing criterion. However, Estelles–Arolas et al. disagree and use the term ‘flexible open call’ meaning that participation is non–discriminatory but the call is tailored to the specific initiative and thus, can be limited to a community where there is specific knowledge or expertise (but anyone in this community can answer) [12].

#### By what medium the call is made

Estelles–Arolas et al. state that of the authors they reviewed, the medium the call was made through was unanimously agreed upon to be the Internet and Estelles–Arolas et al. agreed [12]. However, as stated previously, crowdsourcing has existed prior to the Internet, as has wisdom of the crowds. Thus, while the Internet has enabled crowdsourcing to be used much more effectively and efficiently, it is not necessarily reliant on the Internet as a medium and could be used over a different medium, though this would be less efficient.

### Geiger et al.’s taxonomy/features of crowdsourcing

#### Accessibility of peer contributors

Geiger et al. discuss the degree of which the crowd is able to access each other’s contributions to the product as a feature of the crowdsourcing process and have four categories: none, view, assess or modify [44]. In some crowdsourcing activities, members of the crowd cannot view each other’s contributions at all, while others use a crowd not only to for submissions but also to judge which submissions are the best (ie, Threadless). In other crowdsourcing exercises, participants can modify each other’s submissions. For example, Kittur posted a Spanish poem for translation through crowdsourcing and the crowd was able to interact with each other, discuss possible translations and together, the crowd submitted a final, translated poem. The authors found this translation to be better than the commonly accepted English translation [48]. Finally, Geiger et al. found that some crowdsourcing projects allow the crowd to view other submissions prior to submitting their own [44].

#### Aggregation

Aggregation refers to how the responses of the crowd are used by the crowdsourcer. The two major ways the responses can be used are to be combined or to be selected [44]. InnoCentive, Threadless, and CrowdMed, for example, are *selective* crowdsourcing companies, which choose the best solution or design to a particular problem. Crowdsourcing projects run on AMT often aggregate or combine solutions from the crowd as a whole.

The definition from Estelles–Arolas et al. excludes Wikipedia, YouTube and Flickr. Wikipedia is excluded on the grounds that there is no initiator (crowdsourcing organisation), that the authors do not feel that the initiator receives benefit, and that there is no open call. YouTube is excluded on the grounds that there is no clear goal, that the crowdsourcer’s benefit is not clear, which is arguable as YouTube ‘stars’ receive compensation for views, that there is no clear initiator, the initiator’s benefit is not clearly defined, the crowdsourcing process is not participative and there is no open call. Finally, Flickr, which is a photo sharing website, also fails due to lack of a clear goal, lack of clear benefit to the crowd, lack of clear benefit to the initiator, not being participative and not using an open call [12].

Despite Estelles–Arolas et al.’s integrative definition, some authors strongly believe websites such as Wikipedia are not only examples of crowdsourcing, but are *the classic* examples of crowdsourcing [10]. Indeed, Howe, who ‘coined’ crowdsourcing considers Wikipedia as a classic crowdsourcing example, as do others [4,10,20]. Osella’s review found that some authors’ definitions of crowdsourcing are so expansive that they consider the entire Internet a form of ‘crowdsourcing,’ citing O’Reilly and Batelle: “the Web as a whole is a marvel of crowdsourcing, as are marketplaces such as those on eBay and Craigslist, mixed media collections such as YouTube and Flickr, and the cast personal lifestream collections on Twitter, MySpace, and Facebook” [5].

### When to use crowdsourcing

Many authors reviewed discussed situations that were amenable to crowdsourcing (see **Box 2**). First, crowdsourcing should be used in tasks that require humans, ie, where technology either cannot complete the task or where people can do it better [3,6] and where crowds are better than individuals or experts [6]. But, what specific features would a task need to have to satisfy these broad conditions? Authors have suggested a wide range of conditions which are laid out in **Box 2**. These features are a combination of theoretical and application–based conditions and are, at times, conflicting. Kamajian reviewed crowdsourcing in medicine and his suggestions mirror Surowiecki’s wisdom of the crowds conditions – he believes that the crowd must have tacit knowledge, be diverse but the problem itself must not be tacit, that the firm must not have the knowledge (otherwise why is it seeking the crowd?), and he focuses on the likelihood of the crowd’s expertise and its diversity [14]. In comparison, Kittur describes applications of crowdsourcing, describing those typically conducted through AMT, that are verifiable, have an objectifiably ‘right’ answer, low cognitive load, and require little expertise are most conducive to crowdsourcing [48]. Kamajian’s and Kittur’s images of ideal crowdsourcing are in direct opposition to one another. One feature of ‘when to use crowdsourcing’ that has some agreement is that the task is divisible into lower–level tasks, though this is not a necessary condition [5,48].

Box 2 **Conditions for when to use Crowdsourcing found in review.**

- Tasks that require humans (i.e. where technology either cannot complete the task or where people can do it better) [3,6]
- Crowds are better than individuals or experts [6]
- Firm expertise is low [14]
- Likelihood of crowd expertise is high [14]
- Firm expertise is distant from solution [14]
- Problem is *not* tacit, immobile, unique or complex [14]
- Relevant experience is diverse [14]
- Problem is modular [14]
- Expertise is tacit, immobile, unique or diverse [14]
- IP for problem is protect, problem is not legally protected [14,45]
- No problems with ownership or usage of solution [48]
- Problem does not contain sensitive information [5]
- Problem divisible into lower–level tasks [5,48]
- Low cognitive load [48]
- Problem is fast to complete [48]
- Task requires little expertise [48]
- Solution is objective and verifiable [48]
- Low barriers to entry [48,49]
- Low interaction required [5]

As opposed to focusing on characteristics of projects amenable to crowdsourcing, Buecheler and colleagues describe the characteristics of a principal investigator who would be amenable to taking on a crowdsourcing project. They state that the career age, job satisfaction, cosmopolitan scale, tenure, funding, apparatus and time must be considered; however, the authors do not give an estimate of which features within these characteristics are ideal for crowdsourcing [20].

Other authors gave specific tasks that they felt crowdsourcing was most suitable for, such as solving problems, completing tasks, being creative, developing products or ideas [5]. Castillo believed that crowdsourcing was ideal for medical imaging research, in particular, while Thawrani and colleagues suggested that researchers should use crowdsourcing to capitalise off medical data to find more specific causes of illnesses and also to bring processes up–to–date, such as handwritten medical records in India [13,32].

Finally, some authors reviewed gave tips for using crowdsourcing in research. Most importantly, selecting a clear and appropriate research question was emphasised [2,45,49]. Having a big challenge, and clear, measurable goals that are communicated to participants was seen as important as this helps motivate the participants, along with as providing options regarding levels and modes of participation [49]. Finally, the importance of acknowledging participation was highlighted [49].

### Benefits of using crowdsourcing

Benefits identified in the literature review are divided into process–based benefits and results–based benefits, and are displayed in **Table 2**. Several of these benefits could have fit into both categories. Benefits include the speed of research progression, low cost, increased accuracy of results, ability to coordinate with machine–learning and improve algorithms, act as a public advocacy tool, work in emergency situations, and transcend boundaries and borders. Crowdsourcing is a powerful, flexible tool that can be used in many situations as a supplement to traditional research. Its mobility and low cost make it ideal for global health, where barriers such as lack of human resources, funding, conflict areas and baseline epidemiological data can create barriers to targeting interventions.

**Table 2:** Benefits of crowdsourcing listed by articles reviewed, divided into process–based benefits and results–based benefits

**Process–based benefits**	• Low–cost alternative to traditional behavioural, epidemiological and sensory research [7,19,27,41,48]
	• Large potential scale of participants involved [27]
	• Large scale of coverage of potential intervention [16]
	• Can raise public awareness [27,32,54,55]
	• Transcends borders and boundaries [13]
	• Can be democratic [7]
	• High social robustness [29]
	• High mobility [16]
	• Able to ‘tap into’ untapped expertise [27]
	• Ability to cover unpredictable events [16]
	• Widespread software available to enable feasibility [16]
	• Some benefits difficult to quantify, such as “value of enthusiastic user” [45]
**Results–based benefits**	• Increased accuracy over or when results combined with machine learning tasks [27]
	• Enables high speed of research progression [27,29,48]
	• Novel discoveries [7,27,29,32,48]
	• Data produced previously unattainable [19]
	• Can complete tasks otherwise not possible, including digitizes medical artefacts or notes [32]
	• Rewards may accrue more directly [8]
	• Possible to detect and respond to disease outbreaks earlier [19]
	• Result accuracy has been shown to be equal to or more accurate than traditional research [8,47]
	• Results can improve users’ lives [16]

### Concerns with crowdsourcing

In spite of its benefits, crowdsourcing is still subject to numerous challenges, regulatory, and ethical issues that need to be addressed, considered, and anticipated prior to designing a crowdsourcing study or intervention.

Quality assurance issues were the most commonly identified by the articles reviewed. In instances where a crowd is asked to answer questions where there is no ‘right’ answer, it becomes difficult to verify if responses are true and not malicious [32,48]. Additionally, there is a debate regarding having untrained laypersons complete scientific activities that are normally reserved for experts; experts may protest these activities [8,32]. Finally, concerns were voiced regarding a potential so–called “Hawthorne observer–expected effect,” wherein which members of the ‘crowd’ acts in a way they feel the researcher may want them to [56]. Possible solutions for these issues were proposed, including having multi–level reviews. Here, there are multiple stages to each crowdsourcing task and each task is reviewed multiple times and aggregated [6] having objectifiable tasks to ‘weed out’ malicious workers or having standards by which workers must fulfil prior to be considered for the task [6,27]. For example, in AMT, workers may have obtained certain scores in previous tasks.

Regarding sampling, the denominator is rarely known in crowdsourcing tasks and this can pose problems for analysis [56]. Sampling bias can occur due to inverted sampling [6,8,56] and due to self–reported data [8]. Luan and Law reported cultural and geographical biases in GIS data reviewed [19]. Additionally, there is likely to be biased samples in comparison to the general population with regards to income, literacy, age, access to technology and values [19,56].

Other authors cited concerns for security, citing potential loss of data due to a rise of cyber–attacks [38] or mishandling of sensitive information [32]. Logistical issues cited were specific to platforms or types of crowdsourcing and included troubles with languages and file formats when data mining, trouble with battery life usage, competing with prioritisation of other application on mobile devices, and privacy for ubiquitous computing (sensors in mobile devices) [19] and for AMT, not having proof of payment for work completed and institutional issues gaining approval [3]. In addition, funding being non–traditional was identified as a barrier for all crowdsourcing research [8].

### Regulatory and ethical issues

Despite Thawrani et al.’s and other’s concerns that crowdsourcing could compromise anonymity, other authors were concerned that the anonymity of crowdsourcing could raise ethical concerns [6]. Williams identified instances in which crowdsourcing may have resulted in the deaths of bloggers and could be used to falsely identify (or fail to identify) weapons of mass destruction (WMD) in Iran [6]. As crowdsourcing is a nascent field, there is no Review Ethics Board (REB) or Institutional Review Board (IRB) process specific to it, to the author’s knowledge, despite it being quite different from other methodologies. Exploitation of both the crowdsourcing worker and of the industries the crowdsourcing is taking place in are possible, thus REB/IRB review is very important [7,9,29,56]. Informed consent procedures will differ from general research, as researchers will not have in–person interaction with the participants and will not necessarily be aware of their levels of reading comprehension. The data use policies could represent a unique challenge to informed consent if products are used commercially.

Brabham reports that, while currently it is difficult for crowds to organise themselves against unfair labour practices, “crowdslapping” does happen [7]. This is when a crowd ‘rebels’ against the competition and is, essentially, a crowd of malicious workers, rallying against the project. A recent example of “crowdslapping” is a United Kingdom contest to name an RSS vessel, and the Natural Environment Research Council intended the boat to be named after an inspiring figure. The winning name was “Boaty McBoatface,” which was ultimately rejected in favour of “David Attenborough.” However, a remote undersea vessel was named “Boaty” in memory of the competition [57].

While not considered crowdsourcing by the working definition in this article, text/data mining has unique ethical issues, especially regarding consent, anonymity and researchers planning to use this method must consider this, through community engagement or other methods.

### Notable (non–medical) examples of crowdsourcing

A second paper [58] will review health–related examples of crowdsourcing. Aside from health–related examples, there were over 50 examples of crowdsourcing named in the reviews, with purposes ranging from public policy [42] to mapping isolationist states [6], assisting with or reporting on human rights issues [6,18], mapping or reporting on the environment [6,27], designing t–shirts [1] or linking families [49]. Some notable, interesting and successful examples of crowdsourcing in the non–scientific or medical world are described below:

#### Guardian’s MP expenses

The UK newspaper, the Guardian, utilised crowdsourcing and freedom of information request to have the crowd comb through Members of Parliament’s (MP’s) expense claims to look for fraudulent claims. There were over 500 000 expense claims uploaded and over 170 000 documents were analysed within 80 hours alone [6]. As a result of this activity, British MPs were convicted of fraud, forced to resign or had to issue apologies.

#### Ushahidi

Ushahidi is a SMS– and web–based platform that was created after the Kenyan election in 2007 to report on election violence [6]. It is an open–sourced platform that combines GIS information with time, allowing the crowdsourcing initiator to filter by place and time, which makes it ideal in disaster situations [18]. It has been used for elections, violence, corruption and disasters, including reporting cholera after the Haitian earthquake and in Kenya, Uganda, Nigeria, Haiti, Libya and Egypt [6,53].

#### GalaxyZoo

GalaxyZoo is a crowdsourcing project that uses volunteers from around the globe to classify galaxies visually. As of 2013, it had successfully classified nearly 900 000 galaxies using hundreds of thousands of volunteers [27].

#### Transcribe Bentham

Transcribe Bentham is a project which aims to transcribe works of Jeremy Bentham, a famous utilitarian philosopher, in order for them to be available to all. There were over 12 000 un–transcribed manuscripts and the project is based at University College London (UCL) [59].

#### RECAPTCHA

Captcha stands for “Completely Automated Public Turing test to tell Computers and Humans Apart.” Louis van Ahn, the father of human computing, extended CAPTCHA, adding an additional word so people would need to translate two words; the first was a known ‘anti–bot’ word but the second was from an archive that needed to be digitalised [60]. In 2009, RECAPTCHA was able to digitalise 20 years of the New York Times’ archives and 110 years of archives were projected to be completed by the end of 2010 [60].

## CONCLUSION

Crowdsourcing is a field that is relatively nascent, yet blossoming. Because of its infancy, researchers have not yet agreed on its definition or what does or does not constitute its practice. Despite this, several key qualities have emerged. In order to be considered crowdsourcing, a task must be distributed by an organisation via a flexible open call for the purpose of obtaining some knowledge, idea or added value, through a medium that’s similar but not an outsourced model. Usually, crowdsourcing employs the Internet, though this is not necessary. A crowd can be formed by both experts and amateurs, and the crowd can be rewarded monetarily or through recognition or skill–development. Sometimes the results are aggregated, but in other exercises, the best solution is chosen. In this way, applications of crowdsourcing are themselves very diverse and it is not surprising that authors have struggled to provide an all–encompassing definition.

Despite the difficulties defining it, crowdsourcing is beneficial both in the process and in the results. It is often low–cost, rapid, and has the possibility to transcend fields, borders, can coordinate with machine–learning, raise public awareness and produce novel discoveries. Crowdsourcing could be hugely promising in global health where resources are low and there is a paucity of data if a concerted effort is made to bring it to scale, especially through marrying the global health community with crowdsourcing and computer science researchers.
